# Improving Recruitment Through Social Media and Web-Based Advertising to Evaluate the Genetic Risk and Long-Term Complications in Stevens-Johnson Syndrome and Toxic Epidermal Necrolysis: Community-Based Survey

**DOI:** 10.2196/63712

**Published:** 2025-05-07

**Authors:** Elizabeth A Williams, Michelle D Martin-Pozo, Alexis H Yu, Krystyna Daniels, Madeline Marks, April O'Connor, Elizabeth J Phillips

**Affiliations:** 1 Center for Drug Safety and Immunology, Vanderbilt University Medical Center, 2611 West End Avenue, STE 210, Nashville, TN, United States, 1 615-322-9174; 2 Department of Medicine, Vanderbilt University Medical Center, Nashville, TN, United States; 3 Institute for Immunology and Infectious Diseases, Murdoch University, Perth, Australia

**Keywords:** Stevens-Johnson syndrome, SJS, toxic epidermal necrolysis, TEN, social media, Google Ads, recruitment, diversity, accessibility, rare disease, adverse drug reaction, severe cutaneous adverse reaction, SCAR

## Abstract

**Background:**

Stevens-Johnson syndrome (SJS) and toxic epidermal necrolysis (TEN) are genetically mediated, life-threatening reactions usually caused by a medication in adults. These genetic associations promise an opportunity for pre-prescription screening, prevention, and understanding influences at a population level. Importantly, older adults disproportionally face more severe SJS/TEN reactions and higher mortality rates. However, the study of genetic risk and long-term sequelae of SJS/TEN across racially diverse populations and age groups is hampered by many factors, including rarity, social disparities, and trust in health care and providers, impacting access to hospital- and clinic-based research studies.

**Objective:**

This paper aims to explore the utility of multiple social media and web-based search tools to increase study enrollment numbers, diversity, and inclusivity of all populations and ages in the SJS Survivor Study.

**Methods:**

The community-based SJS/TEN Survivor Study remotely recruited drug-induced SJS/TEN survivors in the United States. The aims were to help determine genetic risk and long-term outcomes of SJS/TEN. Baseline recruitment included advertisements through the SJS Foundation website and American Burn Association newsletter. Two years into the study, in hopes of improving accessibility and enrollment diversity, social media ads were introduced on the Vanderbilt University Medical Center (VUMC) Facebook and Instagram accounts. Posts were created using flyers and 60-second SJS/TEN survivor video vignettes. Finally, we launched a nationwide Google Ad campaign. To understand the impact of the additional online advertising, we measured the change in registration in both the study interest and the effectiveness of implementation of specific social media and web-based search tools before and after implementation.

**Results:**

With the introduction of social media and Google Ads, we report a 48.6% increase in enrollment overall and a 289.5% increase in participation interest. We noticed the ads were accessible to all age groups and notably reported a more even age distribution of enrolled participants from 18 through 74 years, with an average of 15% enrolled in each age category. The largest increase in any age category was seen in the 65‐ to 74-year-old patients (n=19), with 16.5% of the age distribution. The most significant increase in enrollment and diversity of responses came from Google Ads, with a total of 201 expressions of interest, from 56 enrolled participants, 33% of which self-identified as non-White. VUMC Facebook ads had an enrollment rate of 15.3%, and VUMC Instagram ads saw an enrollment rate of 14.3%.

**Conclusion:**

Social media and web-based search tools differ in their enrollment effectiveness. Google Ads were found to be the most effective advertisement for recruitment in this community-based study. Each of the social media and web-based strategies used increased enrollment numbers, accessibility to more age ranges, and diversity of enrollment. They show promise as tools to improve inclusion and enrollment in rare disease research such as SJS/TEN.

## Introduction

Stevens-Johnson syndrome (SJS) and toxic epidermal necrolysis (TEN) are rare, life-threatening drug-induced illnesses affecting 1-5 people per million each year, with mortality rates of 50% or higher in high-risk populations [1,2] SJS/TEN is strongly associated with human leukocyte antigen (HLA) class I alleles, providing an opportunity for preprescription risk prediction, as well as understanding genetic variation across racial and ethnic groups [2,3]. Although studies suggest that the presence of a genetic risk factor has equal implications across populations, the relevance of specific HLA risk alleles may differ due to differences in the HLA carriage rate [3-6]. The study of genetic risk and long-term sequelae of SJS/TEN across racially diverse populations has been burdened by its rarity, social disparities, and trust or access to health care [4,7]. Social media has been advocated as a strategy to improve study diversity and recruitment in rare diseases and increase accessibility for study participation [8,9]. To enhance awareness and enrollment in our community-based study, we implemented social media and web-based advertisements, measuring the utility of each channel in increasing interest and diversity.

## Methods

### Overview

Using historical recruitment data from our community-based study of SJS/TEN survivors, we compared data both before and after implementation of social media and web-based recruitment methods. We recruited SJS/TEN survivors from the United States, aged 7-90 years, to participate in a remote study to assess genetic risk and long-term outcomes in SJS/TEN. Survivors across the United States, who saw or heard about our study, filled out an online interest survey. Data on recruitment and enrollment for this study were collected from September 2019 through December 2023 using the online tool Research Electronic Data Capture (REDCap) and analyzed using Microsoft Excel.

### Recruitment

Baseline recruitment methods included advertisements on the SJS Foundation website and in the American Burn Association newsletter for physician referrals. Two years into the study, enrollment consisted of 147 (72.8%) female participants and 156 (77.2%) participants identifying as non-Hispanic White. In June 2021, we consulted institutional resources to design and implement advertising campaigns across several social platforms to increase interest and diversity. Advertisements were introduced on the Vanderbilt University Medical Center (VUMC) Facebook and Instagram pages using flyers, and 60-second SJS/TEN survivor video vignettes were later added. Next, we launched a nationwide Google Ad campaign. Advertisement-derived interest surveys identified potential participants who were then contacted by phone. Interest surveys reported the mode of recruitment through a single-choice answer of “a website,” “Facebook,” “Instagram,” “Google,” “referral,” or “other.” More detailed responses for choices like “other” were obtained through free response options. Referrals are defined as participants referred to the study by family, friends, or a physician. Ethnicity, race, gender, and age categories were self-reported and followed the National Institute of Health’s reporting guidelines.

To understand the impact of each recruitment channel, we measured rates of study interest before and after the ad implementation, as well as the “enrollment effectiveness” of each channel. Furthermore, these were compared to highlight differences in race, ethnicity, gender, and age.

### Ethical Considerations

The community-based SJS/TEN Survivor Study was approved in August 2019 by the VUMC institutional review board (IRB#191350). Written informed consent was obtained from all enrolled participants. All participants were given the opportunity to withdraw from the study at any time. Participants were given a US $50 Amazon eGift-card as compensation for participation.

Privacy and confidentiality of participants were maintained. All data collected were deidentified before analysis. Data were stored on REDCap, a secure Health Insurance Portability and Accountability Act (HIPAA)-compliant database.

## Results

**Figure 1.**
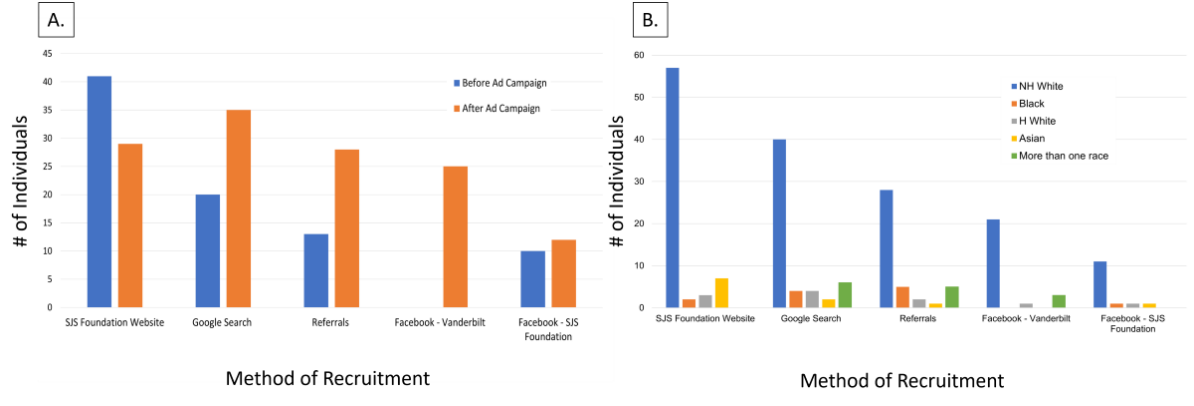
(A) Out of the Stevens-Johnson syndrome and toxic epidermal necrolysis (SJS/TEN) Survivors Study, a community-based survey, we compared how different methods of recruitment affected enrollment. Before advertisement campaigns, enrolled participants self-reported they heard about our study through the SJS Foundation website (n=41, 19.9%), Google search (n=20, 9.7%), referrals (n=13, 6.3%), and the SJS Foundation Facebook website (n=10, 4.9%). With the launch of the advertisement campaigns, the greatest number of enrolled participants heard about the study through Google Ads (n=35, 26.1%). Google was followed closely by the SJS Foundation website (n=29, 21.6%), referrals (n=28, 20.9%), Vanderbilt University Medical Center (VUMC) Facebook ads (n=25, 18.7%), and lastly the SJS Foundation Facebook page (n=12, 9.0%) (**A**). (**B**) The overall enrolled demographic breakdown of self-identified race/ethnicity was 275 (77.9%) non-Hispanic White, 19 (5.4%) Hispanic White, 15 (4.2%) Black, 18 (5.1%) Asian, 1 (0.3%) American Indian, 16 (4.5%) more than one race, and 9 (2.5%) unknown. The greatest enrollment diversity came from referrals, with 15 (36%) identifying as non-White. Google Ads followed with an enrollment diversity of 20 (33%) non-White participants. The SJS Foundation enrollments were 17 (23%) non-White participants, and the VUMC Facebook enrollments were 5 (16%) non-White participants. Google Ads were the most effective strategy across self-identified race and ethnicity. H White: Hispanic White; NH White: Non-Hispanic White.

Prior to implementing ad campaigns, 6.9 unique interest surveys were completed, and 3.4 participants enrolled per month. Following the launch of social media and Google Ads, 26.9 unique interest surveys were completed, and an average of 5 participants enrolled per month, representing a 289.5% increase in interest and a 48.6% increase in enrollments. Ineligibility reasons included lost to follow up (n=253), not diagnosed with SJS/TEN (n=156), duplicate survey (n=73), inability to obtain records (n=65), hospitalized outside of North America (n=38), and declined to participate (n=38; Figure 1).

**Figure 2.**
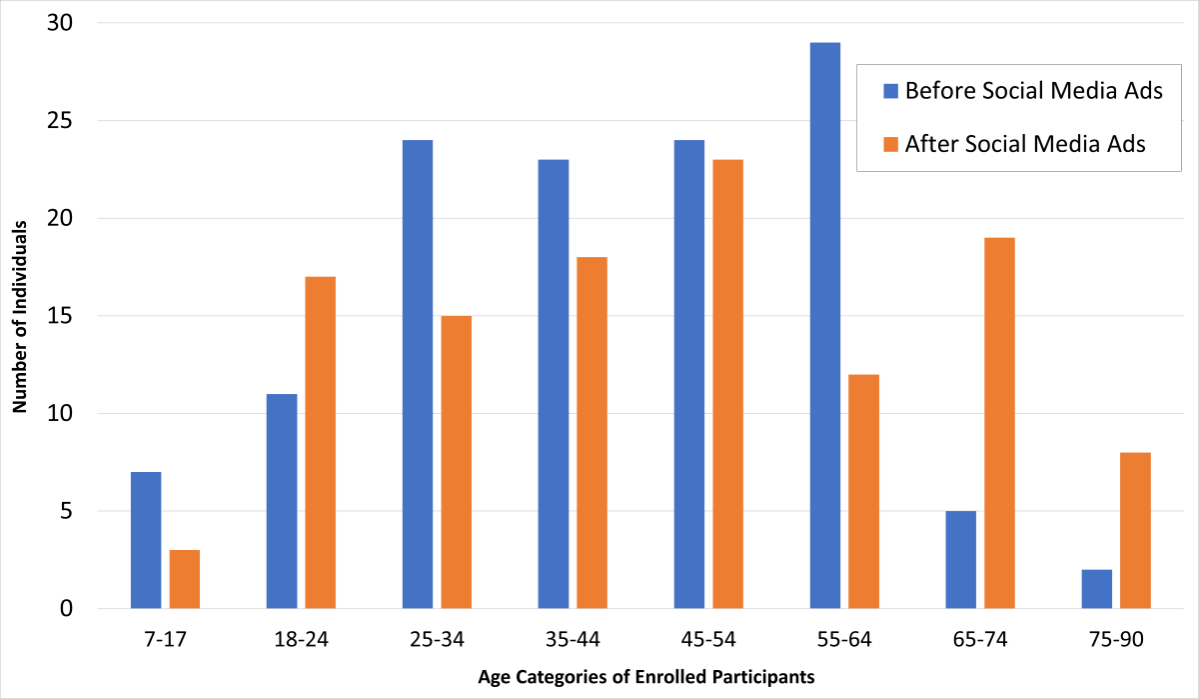
Age breakdown of enrolled participants before and after social media advertisement implementation and the impact of recruitment methods on different age categories. Before social media and Google Ads, we had 60 (14.3%) responses, with 55 (27.2%) enrolled identifying as male. The initial age spread of participants enrolled resembled a bell curve with the highest density of enrollment (n=29, 23%) being between 55 to 64 years. Most enrolled participants (n=100, 80%) were aged 25‐64 years. Following ad campaigns, 131 (21.5%) responses were received, with 16 (17.4%) identifying as male. Notably, more even distribution was seen from 18 through 74 years, with an average of 14.3 (15%) enrolled into each age category. The age categories with growth were 18‐24 (n=17, 14.8%), 65‐74 (n=19, 16.5%), and 75‐90 years (n=8, 7*%).*

Completed interest surveys and enrollment rate were broken down by recruitment channel. The SJS Foundation website produced 149 responses/69 enrolled (46.3% enrollment rate). Google Ads followed with 201 responses/56 enrolled. VUMC Facebook ads contributed 163 responses/25 enrolled (15.3% enrollment rate). Instagram had 7 responses/1 enrolled (14.3% enrollment rate). Participants were also referred by family, friends, and physicians, with 134 responses/41 enrolled (30.6% enrollment rate; Figure 2).

## Discussion

### Principal Findings

Recruitment in studies involving human subjects is often inadequate due to factors including limited patient awareness, lack of trust, and logistical barriers [7-10]. These challenges are frequently compounded when conducting trials on rare diseases like SJS/TEN, partly due to the small number of diagnosed patients. Most studies on SJS/TEN have been country-specific and may not be widely representative of heavily diverse populations like the United States [3,11]. This study is unique as we pulled survivors from the community within the United States, hoping to gain diverse representation. To increase accessibility, enrollment, and knowledge of our trial on SJS/TEN, we implemented several social media-based recruitment methods.

The addition of social media and Google Ads increased interest and enrollment as well as racial, ethnic, and age diversity in recruitment. Google Ads brought in the highest number of responses to the study and the second-highest enrollment rate after the SJS Foundation. Emotional appeals from the survivors’ video testimonials were vital in capturing interest. Supporting this, one study found that after conducting 37 in-depth interviews, patients preferred study recruitment messages through infographics, emotional appeals through images, or educational posts [10]. However, Facebook ads produced inferior responses to Google Ads. Instagram did not gain traction for recruitment, even though the same video testimonials were used.

When evaluating the effectiveness of advertisements on gender, we saw increased male interest, but decreased eligibility and enrollment. Therefore, there was no true impact on gender in terms of enrollment with the increase of social media and web-based advertisements.

An important finding was the increase in responses from people aged over 65 years, in contrast to other studies showing less online engagement from older adults [12]. Older age increases the risk of SJS/TEN-associated morbidity and mortality, making it vital to access older age groups [11,13].

Even with our advertising efforts and the increase in diverse responses, our enrolled participant population remained largely non-Hispanic White females. While a more nuanced understanding is needed, if there is a known HLA allele risk for SJS/TEN for a medication, the implications for carrying the risk allele are the same regardless of country of origin or background [4]. However, the prevalence of different risk alleles does differ by specific population, driven by genetic race. Therefore, the proportion of individuals who develop a reaction to a specific drug can be very population-specific [2,4,5,11]. Given the US population diversity, we need to see more equal representation in SJS/TEN research from all strata in the population to holistically understand the impact of SJS/TEN across the United States.

### Limitations

For the first 10 months of the study, participants were not asked how they heard about our study, resulting in missing recruitment data on 232 unique interest survey responses and 121 enrolled participants. Main recruitment avenues at that time consisted of the SJS Foundation and American Burn Association physician referrals. Another significant limitation is the ongoing cost incurred when using paid advertising. Although we found that social media and Google Ads increased diversity and inclusion, we acknowledge that not everyone can access them. In some cases, older participants will not seek information via the internet [12]. Therefore, exclusive use of web-based search tools could limit accessibility to the study. However, increased diversity associated with referrals and Google Ads supports the use of these advertisements as a valuable source of communicating study information to SJS survivors and their families.

### Conclusions

Our results demonstrated that social media and online recruitment approaches can increase recruitment interest and enrollment in a community-based study of a rare disease such as SJS/TEN while improving inclusivity, diversity, and age ranges. Increasing representation of enrollment across age and different strata of the population supports the unmet need to understand the risk and impact of SJS/TEN on diverse populations. Social media and web-based search tools can differ in enrollment effectiveness and target audiences. Our partnership with the SJS Foundation paired with Google Ads was most effective in reaching SJS/TEN survivors. This study highlights the use of a variety of social media and web-based advertising methods and their potential impact on reaching diverse and hard-to-reach populations. We improved overall enrollment, diversity, and age inclusivity, overcoming recruitment hurdles commonly seen in the rare disease community.

## Abbreviations

HIPAA: Health Insurance Portability and Accountability Act
HLA: human leukocyte antigen
REDCap: Research Electronic Data Capture
SJS: Stevens-Johnson syndrome
TEN: toxic epidermal necrolysis
VUMC: Vanderbilt University Medical Center
